# Imaging adults on extracorporeal membrane oxygenation (ECMO)

**DOI:** 10.1007/s13244-014-0357-x

**Published:** 2014-10-09

**Authors:** Steven Lee, Abhishek Chaturvedi

**Affiliations:** University of Rochester Medical Center, 601 Elmwood Avenue, Box 648, Rochester, NY 14642 USA

**Keywords:** Extracorporeal membrane oxygenation, Cannulas, Thorax, Radiography, CT, Echocardiography

## Abstract

Extracorporeal membrane oxygenation (ECMO) is increasingly being used in adults following failure to wean from cardiopulmonary bypass, after cardiac surgery or in cases of severe respiratory failure. Knowledge of the different types of ECMO circuits, expected locations of cannulas and imaging appearance of complications is essential for accurate imaging interpretation and diagnosis. Commonly encountered complications are malposition of cannulas, adjacent or distal haemorrhage, stroke, stasis thrombus in access vessels, and distal emboli. This article will describe the imaging appearance of different ECMO circuits in adults as well as commonly encountered complications. If a CT (computed tomography) angiogram is being performed on these patients to evaluate for pulmonary embolism, the scan may be suboptimal from siphoning off of the contrast by the ECMO. In such cases, an optimal image can be obtained by lowering the flow rate of the ECMO circuit or by disabling the circuit for the duration of image acquisition.

*Key Points*

• *Femoroatrial VV ECMO: femoral vein drainage cannula and right atrial return cannula.*

• *Femorofemoral VV ECMO: return and drainage cannulas placed in femoral veins.*

• *Dual-lumen single cannula VV ECMO: via the right IJ/Femoral vein with the tip in the IVC/SVC.*

• *Peripheral VA ECMO: peripheral venous drainage cannula and peripheral arterial return cannula.*

• *Central VA ECMO: direct right atrial drainage cannula and aortic return cannula.*

## Introduction

Extracorporeal membrane oxygenation (ECMO) refers to the life support system utilised in pulmonary or cardiopulmonary support for gas exchange [1]. The goal of the system is to oxygenate the patient’s blood while removing carbon dioxide. Also referred to as extracorporeal life support, ECMO is well established in neonatal respiratory failure. Use in adults has increased and ECMO is commonly used following failure to wean from cardiopulmonary bypass after cardiac surgery or in cases of severe respiratory failure [2].

Initial use of ECMO was largely in neonates given findings of increased survival in severely hypoxic infants but no significant benefit to severely ill adults [3, 4]. The results of these initial studies were due in large part to the reversibility of severe respiratory illness in neonates and non-reversible ventilator-associated lung injury present in adults [4]. Today, several inclusionary and exclusionary criteria help guide ECMO application in adults (Table 1).

**Table 1:** Indications and Complications of VA and VV ECMO

TYPE	Indications	Common complications
Central VA	After failure to wean from cardiopulmonary bypass After sternotomy	Mediastinal haemorrhage More invasive May predispose to aortic stasis thrombus
Peripheral VA	When cardiac and pulmonary support is required	Large bore arterial cannulas may predispose to occlusion Malpositioned cannulas Carotid cannulation may contribute to stroke
VV ECMO	Direct support of gas exchange-respiratory failure; ARDS	Malpositioned cannulas DVT in the cannulated limb

Complete discussion of paediatric ECMO is beyond the scope of this article. Neonatal and paediatric ECMO encompasses different cannula sizes, configurations and complications. For example, in neonates arterial cannulation is limited to placement via sternotomy or via cut down technique for carotid cannulation. In such small patients, peripheral cannula placement is exceedingly difficult. Additionally, initiation of VA ECMO usually encompasses carotid or internal jugular vein ligation, a technique that is not well tolerated in adults given the risk of stroke [5]. Similarly, ECMO in the non-neonatal paediatric population may or may not require carotid or jugular ligation. Imaging considerations in paediatric ECMO have been previously discussed in the literature [6]. For the purposes of this discussion, only the imaging of adults on ECMO will be addressed.

In 2009, the CESAR study found improved outcomes in adults treated with ECMO versus conventional therapy [7]. This study randomised patients with severe but reversible respiratory failure to either conventional therapy with intermittent positive pressure ventilation or therapy with ECMO. With the primary end point being severe disability or death at 6 months, the CESAR study demonstrated improved survival and decreased morbidity in patients randomised to ECMO.

There are two types of ECMO: veno-arterial (VA) and veno-venous (VV) [8]. VA ECMO refers to siphoning of deoxygenated blood from a vein with return of oxygenated blood into an arterial vessel. VV ECMO refers to siphoning of deoxygenated blood from a venous vessel and return of oxygenated blood to a systemic venous vessel or the right atrium. The catheters used in ECMO for blood exchange are referred to as ‘cannulas’, a term used in this context to avoid confusion with other vascular catheters. ECMO cannulas may assume variable configurations on radiographs and CTs. Familiarity with differing positions and access sites is important when interpreting images. Additionally, a multitude of complications may arise intrinsic to either ECMO or cannula placement.

## The ECMO circuit

The circuit consists of a pump, blender, oxygenator, control console, heater/cooler and two cannulas. The blender mixes oxygen with CO_2_. This mixture of gas usually consists of 95 % oxygen and 5 % CO_2_ and is referred to as the ‘sweep gas’. The pump is usually a centrifugal or roller pump and is used to transport blood throughout the circuit. The heater/cooler aids in temperature regulation of extracorporeal blood. The cannulas provide direct transport of oxygenated and deoxygenated blood to and from the patient.

The initial oxygenators consisted of venous blood pools that were oxygenated with oxygen bubbles. These first clinical attempts at extracorporeal life support began in the 1950s and were largely unsuccessful beyond several hours as direct exposure of blood to oxygen led to life-threatening coagulopathy, haemolysis, and multi-organ failure. In 1959, Burns developed the definitive solution involving a silicone membrane that separated blood from oxygen and allowed for indirect oxygenation of blood [9]. This was a major step in extracorporeal life support. In the years following (1972–1976) the first published reports of the successful use of extracorporeal life support emerged [9]. Modern membranes are largely made from polymethyl-pentene, which is lower in resistance and causes less consumption of blood products [10].

### Indications for initiation of adult ECMO

VA ECMO is used for both cardiac and pulmonary support such as acute cardiac failure or failure to wean from cardiopulmonary bypass after cardiac surgery [11]. VV ECMO is used for reversible respiratory failure with normal cardiac function. The most common use is in acute respiratory distress syndrome, which may be secondary to severe pneumonia or influenza [11]. More specifically, eligibility was defined in the CESAR trial by a Murray score >3, a pH of less than 7.20 and having a reversible disease process.

**Fig. 1:**
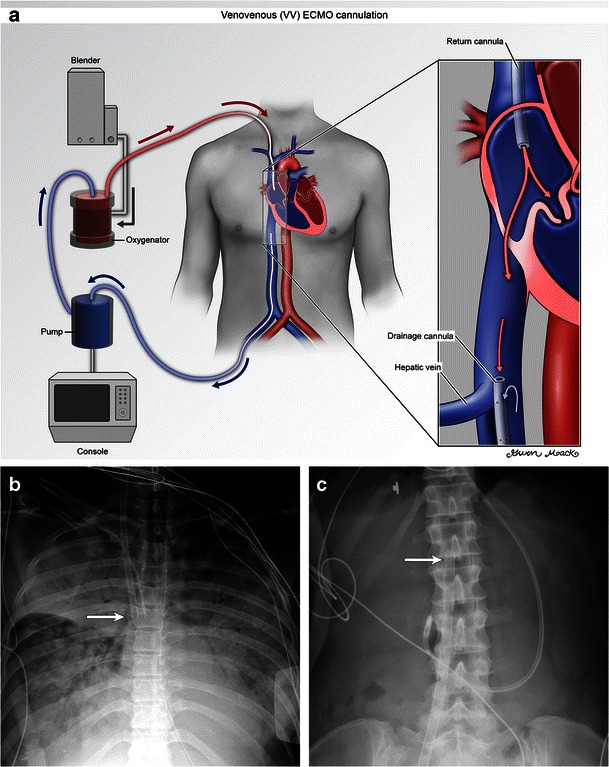
Illustration (**a**) and radiograph (**b**) of femoroatrial VV ECMO demonstrate a right femoral drainage cannula and right atrial return cannula. Frontal radiographs show the expected locations of the right atrial return cannula (b, arrow) and right femoral drainage cannula (c, arrow). While the Murray score depends on several factors, this patient would receive a 4 for the chest radiograph element as there is four-quadrant infiltration

The Murray score consists of four factors including (1) the PaO_2_/FiO_2_ ratio, (2) PEEP (positive end expiratory pressure), (3) dynamic lung compliance in ml/cmH_2_0 and (4) number of quadrants infiltrated on radiographs [7] (Fig. 1b). Each element is graded on a scale of 0–4. The scores are then averaged and the mean score is used to grade the severity of respiratory disease; 0 implies no significant disease and 4 implies the most severe disease. Normal for the above elements are PaO_2_/FiO_2_ ≥ 40 kPa, PEEP ≤ 5, compliance ≥ 80 and 0 quadrants infiltrated on the radiograph [12].

### Contraindications to ECMO

Relative contraindications include high pressure ventilation, high FiO_2_, limited vascular access and organ dysfunction that would lead to poor quality of life. In patients with pre-existing disease such as metastatic cancer or severe irreversible brain injury, ECMO may be contraindicated. Absolute contraindications include any conditions that preclude anticoagulation [8].

### Imaging modalities used in evaluation of ECMO

Transthoracic echocardiography (TTE) and transesophageal echocardiography (TEE) play crucial roles during initial cannula placement. A full discussion on the role of echocardiography in ECMO is discussed by Platts et al. in “The Role of Echocardiography in the Management of Patients Supported by Extracorporeal Membrane Oxygenation” [13].

Radiographs of the chest and abdomen are useful initial examinations for cannula position. They may reveal malposition or unintended migration. They often offer the first clue towards complications such as haemothorax, pneumothorax or mediastinal fluid collections.

Ultrasound is a portable, focussed and readily available modality that can be used to evaluate for insertion site haematomas, peri-cannula thrombus or deep venous thrombus. Spectral colour Doppler is a powerful tool to evaluate distal limb perfusion as the arterial return cannula can lead to obstruction/occlusion with decreased forward flow in the access artery. It can also be used for evaluating thrombus in the access vein. Interpretation of spectral Doppler waveforms with comparison of flow in the contralateral vessel can help in differentiating slow flow, which may be due to a systemic cause such as poor cardiac output, from slow flow in the access artery from either the access cannula itself or thrombus.

CT is reserved for evaluation of complications that cannot be fully evaluated by radiographs, ultrasound or Doppler imaging. While lacking the functional data of spectral Doppler, CT provides excellent anatomic detail. This may be used in evaluating cannula position or malposition, haematoma formation, haemothorax, stroke or arterial/venous thrombus in large vessels. A full summary of the different imaging modalities and their indications are provided in Table 2.

**Table 2:** Imaging modalities in ECMO

Imaging modality	Uses	Key characteristic
Radiograph	Cannula positioning Pneumothorax and haemothorax Evaluation of quadrants for Murray scoring.	Initial study Widely available Comparison to priors for change
Echocardiography	During initial placement of ECMO cannulas	Evaluate recovery of cardiac function
Ultrasound ± Doppler	Evaluation of vascular patency Haemothorax Haematoma at cannulation site	Comparison of cannulated and non-cannulated vessels aids in evaluation of abnormal vascular waveforms
NCCT	Malpositioned cannulas Haematoma Stroke Pulmonary/Abdominal infection Aortic stasis thrombus	Maintain resting respiratory rate to minimise motion artefact
CECT	Pulmonary stasis thrombus Aortic stasis thrombus	Switch ECMO circuit to minimal flow status or stop the ECMO pump for the duration of the acquisition

*NCCT* non-contrast computer-aided tomography, *CECT* contrast-enhanced computer aided tomography

### VV ECMO

VV ECMO is usually placed percutaneously and peripherally. Several configurations are seen. In the ‘femoroatrial’ configuration the drainage cannula is introduced via the femoral vein and advanced to the level of the diaphragm, below the hepatic veins [13]. The return cannula is introduced via the internal jugular vein and advanced to the level of the superior vena cava (SVC)-atrial junction (Fig. 1). The tip is directed towards the tricuspid valve. This particular configuration is optimal to minimise recirculation [14], a phenomenon in which a portion of the returning oxygenated blood is prematurely siphoned back to the ECMO circuit. Recirculation will result in less oxygenated blood returning to the pulmonary circulation and thus the systemic circulation.

A configuration in which the drainage cannula and return cannula are introduced via the femoral veins is termed 'femorofemoral ECMO'. In this configuration the drainage cannula may be placed on one side and the return cannula on the other. Alternatively, both cannulas may be introduced on the same side. In either case the drainage cannula is advanced to the distal inferior vena cava (IVC) and the return cannula is advanced to the right atrium [14].

**Fig. 2:**
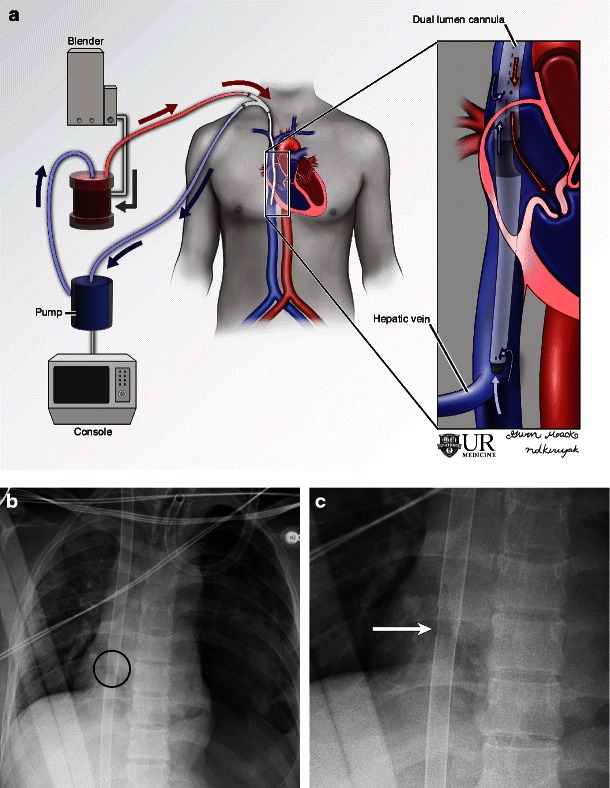
Illustration (**a**) and radiograph (**b**) of dual lumen, single cannula VV ECMO demonstrate the drainage cannula tip in the IVC and a sidehole in the right atrium, which is used for blood return. A magnified view of the right atrium and cannula (**c**) demonstrates the atrial opening identified by the slight discontinuity of the cannula (*arrow*)

Another configuration of VV ECMO is the single cannula, dual lumen ECMO (Fig. 2a) [14]. This cannula is usually inserted via the right internal jugular vein with the tip advanced to the IVC. Alternatively this cannula may be inserted via the femoral vein with the tip advanced to the SVC. The atrial return port is identified on radiographs as a lucency in the cannula (Fig. 2b, c). Rarely, this dual lumen cannula may be inserted via the left internal jugular vein if the right is scarred or thrombosed. The distal tip of this cannula drains deoxygenated blood and a separate opening at the level of the right atrium allows for oxygenated blood return.

### VA ECMO

Compared with VV ECMO, the VA variation is associated with a higher incidence of bleeding, a greater need for transfusion, an increased need for reoperation and greater resource utilisation [15]. Despite this, VA ECMO is preferred when cardiac support is required in addition to pulmonary support and also after sternotomy. The two main types are central and peripheral. Central refers to cannula placement in the mediastinum and peripheral VA ECMO refers to placement outside of the mediastinum [13]. A summary comparison between VA and VV ECMO can be found in Table 3.

**Table 3:** Summary of common ECMO configurations

Optimal cannula tip position	Drainage cannula	Return cannula	Key characteristics
VA ECMO			
Central	Right atrium	Aorta	Direct insertion into the mediastinal vessels
Peripheral	Distal IVC or SVC, before the cavoatrial junction	Proximal femoral artery, axillary artery, subclavian artery	Insertion of cannulas in peripheral vessels
VV ECMO			
Femorofemoral	Distal IVC, at the level of the diaphragm	Right atrium via the same or opposite iliofemoral vein	Less recirculation and improved flow
Femoroatrial	Distal IVC, at the level of the diaphragm	Distal SVC/right atrium via the SVC	Optimal to minimise recirculation
Dual lumen, single cannula	IVC, below the diaphragm	Right atrium via the SVC	For urgent pulmonary support

*IVC* inferior vena cava, *SVC* superior vena cava, *DVT* deep vein thrombus

**Fig. 3:**
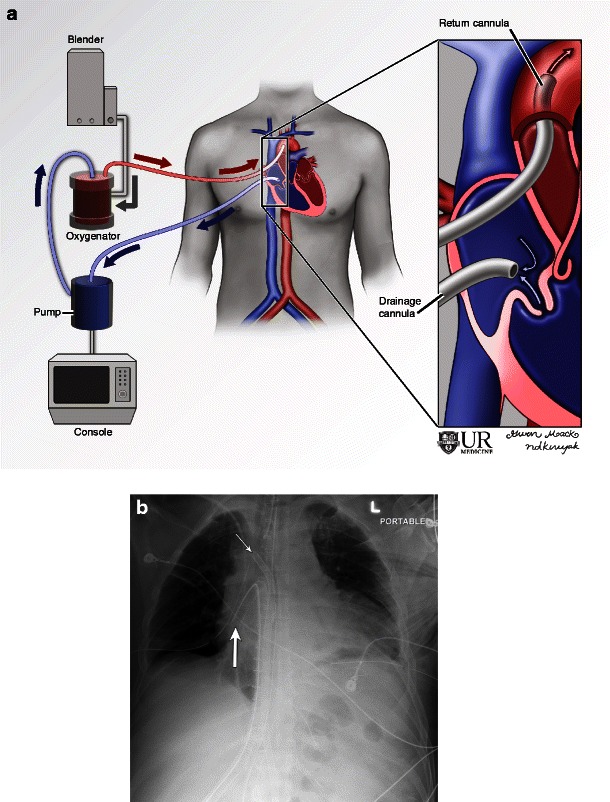
Illustration (**a**) and radiograph (**b**) of mediastinal (central) VA ECMO. Note the drainage cannula in the right atrium (*thick arrow*) and the return cannula in the ascending aorta (thin arrow)

**Fig. 4:**
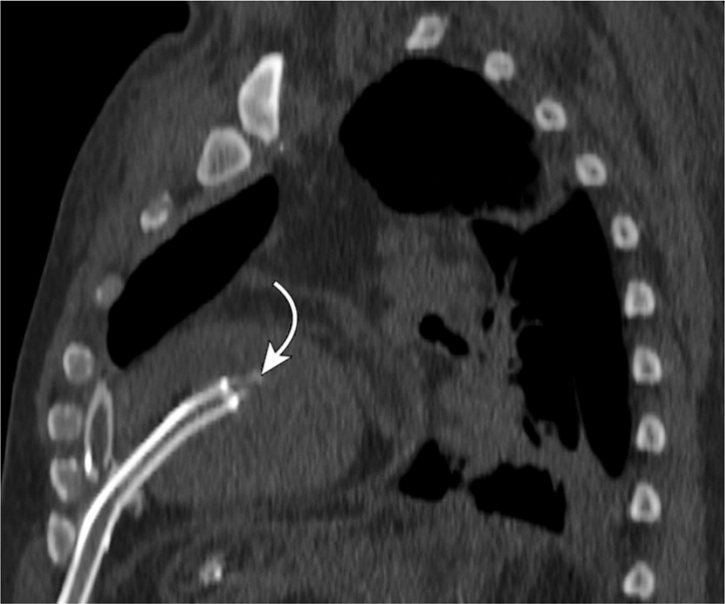
Sagittal reformat from a CT demonstrating a left ventricular drain (arrow) terminating in the left ventricle. This drain aids in decompression when left ventricular function is poor

Also known as mediastinal VA ECMO, central VA ECMO involves direct placement of cannulas into the aorta (return cannula) and right atrium (drainage cannula) through an open sternum (Fig. 3). This is usually done when there is failure to wean from cardiopulmonary bypass in the operating room as direct access is readily available. If left ventricular (LV) function is poor, a drainage catheter may be placed in the LV (Fig. 4) to aid in decompression [16]. Left ventricular unloading is indicated when there is an extremely poor ejection fraction with a closed aortic valve [16]. This predisposes the patient to stasis of blood in the ventricle and subsequently thrombus formation. Patients with aortic regurgitation may also have a LV drain as retrograde flow of blood may compromise ventricular recovery after the initial insult [16]. Finally, patients with severe pulmonary oedema may also require an LV drain to help decrease the pulmonary afterload [16]. The drain can be introduced directly into the left ventricle after sternotomy. Alternatively, the LV drain can also be introduced via the femoral artery or right upper pulmonary vein. The LV drain is connected to the ECMO circuit along with the drainage cannula.

**Fig. 5:**
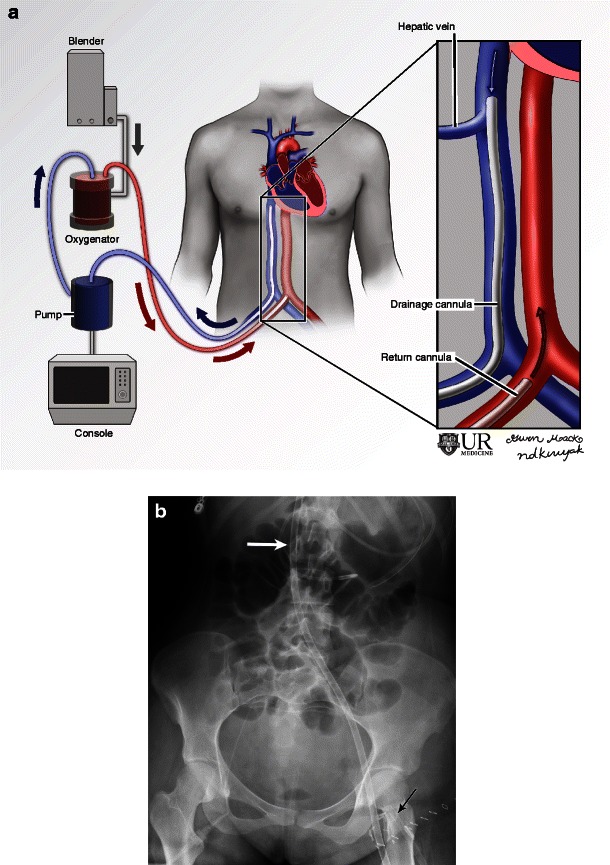
Illustration (**a**) and radiograph (**b**) demonstrating peripheral VA ECMO. The drainage cannula is advanced in the distal IVC to the level of the diaphragm. The arterial return cannula is not advanced very far. Radiograph (**b**) demonstrating the arterial cannula (black arrow) in the proximal femoral artery with multiple surgical clips used for anchoring. The full extent of the venous drainage cannula (white arrow) can be better assessed with a chest radiograph (not shown)

**Fig. 6:**
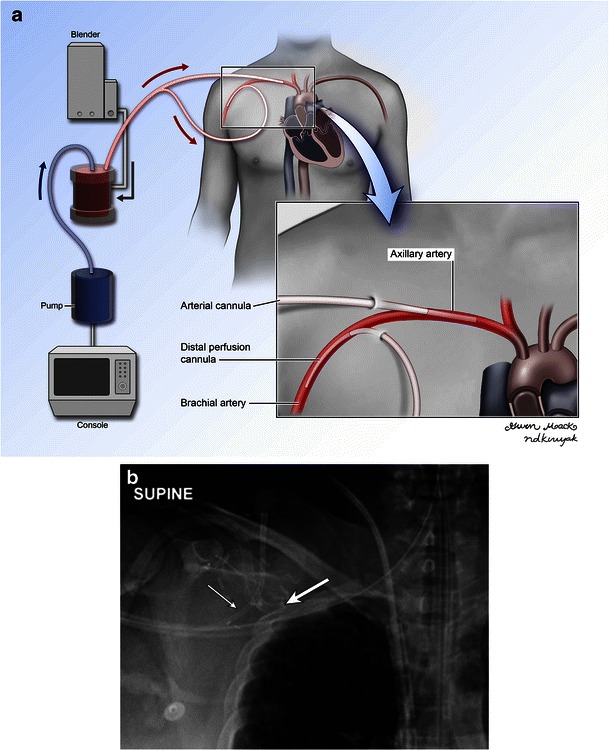
Illustration (**a**) and radiograph (**b**) demonstrating a distal arterial perfusion cannula (thin arrow) in a patient on peripheral VA ECMO. The arterial return cannula (thick arrow) projects over the axillary artery and points proximally as compared to the perfusion cannula which points distally

In peripheral VA ECMO, the arterial cannula may be seen projecting over the femoral or axillary arteries. At our institution, carotid artery cannulation is less commonly used in adults. The arterial cannulas are usually advanced to the level of the iliac arteries or abdominal aorta [13], and usually multiple surgical clips are used for anchoring that are readily visualised on radiographs. The venous cannulas may be seen on the same side as the arterial cannula but are advanced closer to the right atrium, terminating in the SVC or IVC (Fig. 5). Often a distal perfusion catheter is placed in the access artery to prevent distal perfusion defects and limb ischaemia (Fig. 6).

## Complications

### Cannula malposition

**Fig. 7:**
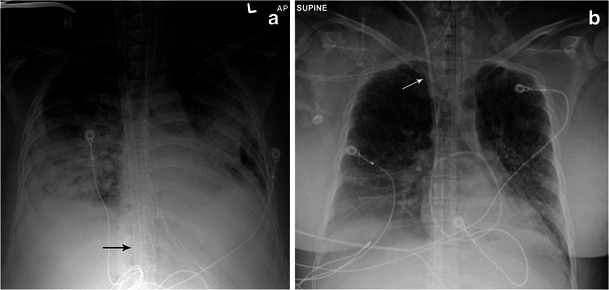
Malpositioned cannulas. Chest radiograph of a 54-year-old male status post MI on peripheral VA ECMO **a** demonstrates the tip of a superior approach venous ECMO cannula projecting too inferiorly over the IVC (*thin arrow*). A chest radiograph from a different patient **b** shows the tip of an inferior approach venous cannula projecting over the lower SVC, much higher than the expected location of the right atrium

Initial placement of ECMO cannulas is usually confirmed by echocardiography and the position reaffirmed by radiographs. The cannulas are anchored by sutures to prevent displacement or malposition. Any change in the position of the cannulas on follow-up radiographs should be noted. As noted above, for VV ECMO, venous drainage cannulas should be placed in the proximal SVC or IVC and return cannulas should be placed in the right atrium. Although discerning artery from vein may be difficult on radiographs, any change in cannula positioning with reference to adjacent bony landmarks should prompt the radiologist to enquire about ECMO malfunction. If suspected, CT and ultrasound are better suited to evaluate cannula dislodgement and/or displacement. A superior approach cannula placed too inferiorly may traverse the right atrium into the IVC and obstruct hepatic outflow (Fig. 7a). Alternatively, an inferior approach cannula placed too high may obstruct the SVC (Fig. 7b).

### Gas emboli

Gaseous microemboli can be introduced into the ECMO circuit during cannula placement, from inadequate priming of the filter system, from the perfusionist accessing the circuit (for blood draws or medication injection), from the pump or from turbulent flow within the tubing [17, 18]. Only large air emboli may be seen on conventional imaging and only a few cases exist in the literature. Gaseous microemboli are not well seen on conventional imaging and in fact require specialised ultrasound machines for detection in the circuit [17].

### Access vessel obstruction/occlusion

Vascular complications are seen in 10–16.9 % of patients on ECMO [19]. Clinical presentation may include pallor, diminished or absent pulses, compartment syndrome or gangrene. In our experience, spectral Doppler ultrasound is the imaging modality of choice for evaluation of peripheral vessels in this setting.

**Fig. 8:**
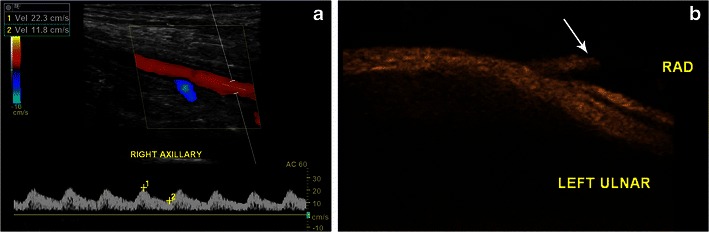
Arterial vascular obstruction. Spectral Doppler image of the right axillary artery (**a**) in a 42-year-old female on VA ECMO demonstrates diminished amplitude and prolonged systolic peaks of the arterial waveforms, classic for pulsus parvus et tardus. In a different patient, B flow ultrasound (**b**) shows complete occlusion of the radial artery (*arrow*) just distal to the bifurcation

The large size of arterial cannulas poses a significant risk of occlusion [20]. Initial examinations with spectral Doppler in the artery distal to the cannula may demonstrate an arterial waveform with a prolonged systolic peak and diminished amplitude (pulsus parvus et tardus) (Fig. 8a). However, it is normal for the peak and mean velocities to decrease by 30–50 % after ECMO placement [21]. Occlusion may also show lack of flow as well as absent waveforms (Fig. 8b). Given the risk of occlusion, some surgeons may choose to place arterial catheters in the distal extremity to augment downstream limb perfusion. Options include placement of an anterograde catheter in the common femoral artery/axillary artery or placement of a retrograde catheter in the tibial artery [22, 23]. It is important to note that vascular waveforms also depend on myocardial function, particularly left ventricular function. A poor ejection fraction may also produce dampened waveforms or accentuate partial occlusion by a cannula. This can be assessed by comparing waveforms in the contralateral limb.

In venous obstruction, colour Doppler may show a lack of flow or absence of normal phasicity on spectral Doppler imaging. Although much consideration is given to arterial perfusion, venous drainage should be an equally important consideration. If the venous cannula is large enough to obstruct venous return, severe limb oedema and ischaemia result [23]. As such, one may see an additional catheter in the distal access vein that assists with venous drainage. Comparing flow in the non-cannulated extremity vessel will again help elucidate whether the suspected flow abnormality is due to poor cardiac function or true occlusion.

### Venous and arterial thrombus

The incidence of deep venous, pulmonary and arterial thrombosis in patients on ECMO is not well documented in the literature. In fact experience with these entities is restricted mainly to case reports. In cases of acute arterial thrombus in the setting of peripheral VA ECMO, spectral Doppler imaging will show lack of flow, absent waveforms and an abrupt cutoff of distal vessels. In venous occlusion by thrombus, spectral Doppler imaging may show thrombus within or surrounding the cannula. There may also be enlargement of the affected vein and extension of the thrombus distally.

**Fig. 9:**
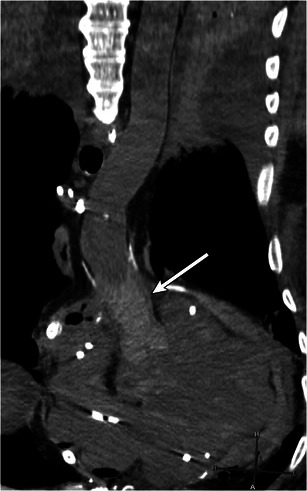
Arterial stasis thrombus. Non-contrast reconstructed CT image in a 67-year-old male on VA ECMO after a failed heart transplant demonstrates intraluminal high attenuation material in the proximal tubular ascending aorta compatible with thrombus

With poor left ventricular function in patients on VA ECMO, inadequate flow may lead to stasis thrombus forming in the left ventricle, left ventricular outflow tract and ascending aorta proximal to the return cannula. Non-contrast CT will demonstrate high-density thrombus in the ascending aorta, proximal to the insertion of the arterial cannula (Fig. 9). Additionally, similar cases of thrombus forming in the aortic root and descending aorta have been described in the literature but are rare [24]. Common clinical presentations are biventricular failure and failure to wean from ECMO. Not surprisingly, case reports also document significant mortality.

### Cerebral ischaemia and stroke

**Fig. 10:**
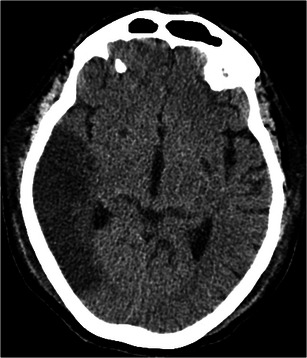
Stroke in a 48-year-old male on VA ECMO with mental status change. CT of the head demonstrates a large hypodensity in the distribution of the right MCA compatible with subacute infarct

Up to 50 % of patients on ECMO demonstrate severe neurological sequelae [25]. It is however unclear whether neurological damage is secondary to ECMO itself or secondary to the patients inciting reason for hospitalisation [25]. Autopsies performed on patients who did not survive ECMO demonstrated evidence of hypoxic-ischaemic injury or injury in a vascular distribution [25]. The clinical suspicion for stroke may be obscured in ECMO patients given the multitude of other systemic or metabolic derangements usually encountered in ICU patients. For new-onset neurological symptoms, CT may be used to identify cerebral infarcts (Fig. 10).

### Haemorrhage

**Fig. 11:**
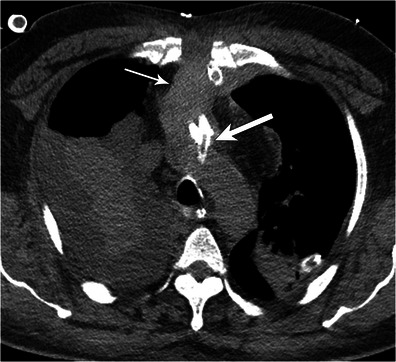
Haematoma. Axial CT in a 44-year-old male on mediastinal ECMO for heart failure after myocardial infarction shows mediastinal haematoma (thin arrow) tracking along the arterial cannula (thick arrow) up to the aortic arch

Haemorrhage can occur at cannulation insertion sites as a direct result of puncture or distally due to coagulopathy. For central VA ECMO the earliest clue will be new widening of the cardiomediastinal silhouette on the chest radiograph. Haematoma is better appreciated on CT as a high-attenuation collection adjacent to the cannula (Fig. 11). For peripheral VA ECMO, ultrasound may show a heterogeneous collection surrounding an echogenic cannula

Distal haematoma may be seen as haemothorax or haemorrhagic ascites. It is usually multifactorial. Factors such as anticoagulation, consumption of complement and prothrombotic substances in addition to recent cannulation may cause subacute haemothorax to develop over time. Radiographs may demonstrate new effusion and/or displacement of mediastinal structures. Ultrasound will demonstrate echogenic fluid that can be confirmed by CT as high-attenuation fluid.

## Optimizing CT angiogram for pulmonary emboli

**Fig. 12:**
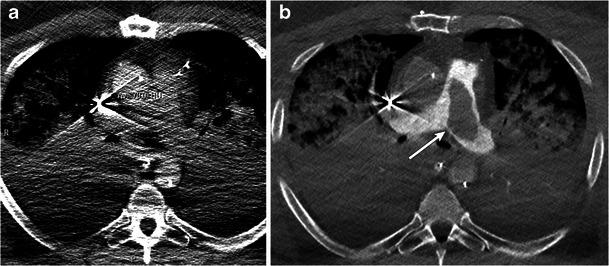
Pulmonary embolism. Axial CT image of a 44-year-old male on central VA ECMO **a** shows insufficient opacification of the pulmonary arteries using the standard protocol secondary to siphoning of IV contrast by ECMO. Diagnositc images are obtained in the same patient **b** after putting the circuit in a minimal flow state (500 ml/min) for 15 s with reinjection of IV contrast. Thrombus is seen in the main pulmonary artery (b, arrow)

Poor right ventricular function will predispose patients to slow flow within the pulmonary arterial system and thrombus formation. Evaluating these patients remains a challenge. As intra-venous contrast is injected, the venous cannula of the ECMO circuit siphons off contrast before it can opacify the pulmonary arteries [26]. Evaluation of pulmonary embolism using standard protocols may lead to suboptimal opacification of the main pulmonary artery and a non-diagnostic examination (Fig. 12a). If clinical suspicion for pulmonary embolism remains high and initial CT angiography with the ECMO circuit fully operational leads to a non-diagnostic examination, the solution may be obtained in two ways.

The first is to reduce pump flow to the ECMO circuit typically to 500 cc/min for 15–25 s, inject IV (intravenous) contrast and place the region of interest locator at the main pulmonary artery. Minimal flow will preclude thrombus formation in the cannulas. With the ECMO circuit in low-flow status, IV contrast will opacify the pulmonary arteries [27]. As cardiac function may be poor in these patients, CT acquisition may be manually triggered when the region of interest demonstrates a peak or a plateau in the opacification of the main pulmonary artery. This plateau will vary according to right ventricle function and may be different for each patient. Triggering the scan will vary accordingly. Scan parameters are as follows: kVp and mAs are as determined from the initial scouts. IV contrast is injected at 4–5 cc/s (contrast volume = 4–5 cc × [delay time of the scanner from initiation of bolus trigger + scan duration]) [28].

If pulmonary arterial opacification is suboptimal with the above technique, a repeat scan should be considered with the circuit completely disabled for the duration of the CT acquisition. This will typically be 10–15 s for a 64-slice MDCT. The pulmonary arteries will be opacified in a near anatomic manner depending on cardiac function. Bolus tracking as described above may be used.

## Conclusion

ECMO cannula configurations are extremely variable. The radiologist should be familiar with the expected locations of cannulas in VV ECMO, peripheral VA ECMO and central VA ECMO. As such, the radiologist should be able to comment on malposition and prospectively search for complications such as vascular obstruction. Additionally, there should be a high index of suspicion for haemorrhage, venous thrombus, stasis thrombus and cerebral ischaemia. Familiarity with the appropriate imaging modalities and common complications will be useful for the interpreting radiologist.

## References

[1] Allen S (2011). A review of the fundamental principles and evidence base in the use of extracorporeal membrane oxygenation (ECMO) in critically ill adult patients. J Intensive Care Med.

[2] Organization ELS (2014) ELSO website, under registry information. [website] 2014 [cited 2014; Available from: http://www.elso.org/index.php?option=com_content&view=article&id=95&Itemid=478.

[3] Zapol WM (1979). Extracorporeal membrane oxygenation in severe acute respiratory failure. A randomized prospective study. JAMA.

[4] Maslach-Hubbard A, Bratton SL (2013). Extracorporeal membrane oxygenation for pediatric respiratory failure: history, development and current status. World J Crit Care Med.

[5] Fenik JC, Rais-Bahrami K (2009). Neonatal cerebral oximetry monitoring during ECMO cannulation. J Perinatol.

[6] Goodwin SJ (2014). Chest computed tomography in children undergoing extra-corporeal membrane oxygenation: a 9-year single-centre experience. Pediatr Radiol.

[7] Peek GJ (2009). Efficacy and economic assessment of conventional ventilatory support versus extracorporeal membrane oxygenation for severe adult respiratory failure (CESAR): a multicentre randomised controlled trial. Lancet.

[8] Brodie D, Bacchetta M (2011). Extracorporeal membrane oxygenation for ARDS in adults. N Engl J Med.

[9] Lim MW (2006). The history of extracorporeal oxygenators. Anaesthesia.

[10] Khoshbin E (2005). Performance of polymethyl pentene oxygenators for neonatal extracorporeal membrane oxygenation: a comparison with silicone membrane oxygenators. Perfusion.

[11] Sidebotham D (2009). Extracorporeal membrane oxygenation for treating severe cardiac and respiratory disease in adults: Part 1–overview of extracorporeal membrane oxygenation. J Cardiothorac Vasc Anesth.

[12] Murray JF (1988). An expanded definition of the adult respiratory distress syndrome. Am Rev Respir Dis.

[13] Platts DG (2012). The role of echocardiography in the management of patients supported by extracorporeal membrane oxygenation. J Am Soc Echocardiogr.

[14] Sidebotham D (2010). Extracorporeal membrane oxygenation for treating severe cardiac and respiratory failure in adults: part 2-technical considerations. J Cardiothorac Vasc Anesth.

[15] Kanji HD (2010). Peripheral versus central cannulation for extracorporeal membrane oxygenation: a comparison of limb ischemia and transfusion requirements. Thorac Cardiovasc Surg.

[16] Rupprecht L (2013). Cardiac decompression on extracorporeal life support: a review and discussion of the literature. ASAIO J.

[17] Lou S (2011). Generation, detection and prevention of gaseous microemboli during cardiopulmonary bypass procedure. Int J Artif Organs.

[18] Win KN, Wang S, Undar A (2008). Microemboli generation, detection and characterization during CPB procedures in neonates, infants, and small children. ASAIO J.

[19] Cheng R (2014). Complications of extracorporeal membrane oxygenation for treatment of cardiogenic shock and cardiac arrest: a meta-analysis of 1,866 adult patients. Ann Thorac Surg.

[20] Rao AS (2010). A novel percutaneous solution to limb ischemia due to arterial occlusion from a femoral artery ECMO cannula. J Endovasc Ther.

[21] Chauhan S, Subin S (2011). Extracorporeal membrane oxygenation, an anesthesiologist’s perspective: physiology and principles. Part 1. Ann Card Anaesth.

[22] Kasirajan V (2002). Technique to prevent limb ischemia during peripheral cannulation for extracorporeal membrane oxygenation. Perfusion.

[23] Russo CF (2009). Prevention of limb ischemia and edema during peripheral venoarterial extracorporeal membrane oxygenation in adults. J Card Surg.

[24] Madershahian N et al (2013) Thrombosis of the aortic root and ascending aorta during extracorporeal membrane oxygenation. Intensive Care Med 10.1007/s00134-013-3173-8 24306084

[25] Mateen FJ (2011). Neurological injury in adults treated with extracorporeal membrane oxygenation. Arch Neurol.

[26] Auzinger G et al (2013) Computed Tomographic Imaging in Peripheral VA-ECMO: Where Has All the Contrast Gone? J Cardiothorac Vasc Anesth 10.1053/j.jvca.2013.06.027 24183316

[27] Liu KL (2014). Multislice CT scans in patients on extracorporeal membrane oxygenation: emphasis on hemodynamic changes and imaging pitfalls. Korean J Radiol.

[28] Khadir MM (2014). Looking beyond the thrombus: essentials of pulmonary artery imaging on CT. Insights Imaging.

